# Toll-like receptor signaling adapter proteins govern spread of neuropathic pain and recovery following nerve injury in male mice

**DOI:** 10.1186/1742-2094-10-148

**Published:** 2013-12-09

**Authors:** Jennifer A Stokes, Jonathan Cheung, Kelly Eddinger, Maripat Corr, Tony L Yaksh

**Affiliations:** 1 Department of Pharmacology, University of California, 9500 Gilman Dr. MC 0636, La Jolla, San Diego, CA 92093-0636, USA; 2 Department of Anesthesiology, University of California, 9500 Gilman Dr. MC 0818, La Jolla, San Diego, CA 92093-0818, USA; 3 Division of Rheumatology, Allergy and Immunology, University of California, 9500 Gilman Dr. MC 0663, La Jolla, San Diego, CA 92093-0663, USA

**Keywords:** Allodynia, Hyperalgesia, Interferon-beta, MyD88, Spinal nerve ligation, Toll-like receptors, TRIF

## Abstract

**Background:**

Spinal Toll-like receptors (TLRs) and signaling intermediaries have been implicated in persistent pain states. We examined the roles of two major TLR signaling pathways and selected TLRs in a mononeuropathic allodynia.

**Methods:**

L5 spinal nerve ligation (SNL) was performed in wild type (WT, C57BL/6) male and female mice and in male *Tlr2*^*-/-*^*Tlr3*^*-/-*^, *Tlr4*^*-/-*^, *Tlr5*^*-/-*^, *Myd88*^*-/-*^, *Trif*^*lps2*^, *Myd88/Trif*^*lps2*^, *Tnf*^*-/-*^, and *Ifnar1*^*-/-*^ mice. We also examined L5 ligation in *Tlr4*^*-/-*^ female mice. We examined tactile allodynia using von Frey hairs. Iba-1 (microglia) and GFAP (astrocytes) were assessed in spinal cords by immunostaining. Tactile thresholds were analyzed by 1- and 2-way ANOVA and the Bonferroni *post hoc* test was used.

**Results:**

In WT male and female mice, SNL lesions resulted in a persistent and robust ipsilateral, tactile allodynia. In males with TLR2, 3, 4, or 5 deficiencies, tactile allodynia was significantly, but incompletely, reversed (approximately 50%) as compared to WT. This effect was not seen in female *Tlr4*^*-/-*^ mice. Increases in ipsilateral lumbar Iba-1 and GFAP were seen in mutant and WT mice. Mice deficient in MyD88, or MyD88 and TRIF, showed an approximately 50% reduction in withdrawal thresholds and reduced ipsilateral Iba-1. In contrast, TRIF and interferon receptor null mice developed a profound ipsilateral and contralateral tactile allodynia. In lumbar sections of the spinal cords, we observed a greater increase in Iba-1 immunoreactivity in the TRIF-signaling deficient mice as compared to WT, but no significant increase in GFAP. Removing MyD88 abrogated the contralateral allodynia in the TRIF signaling-deficient mice. Conversely, IFNβ, released downstream to TRIF signaling, administered intrathecally, temporarily reversed the tactile allodynia.

**Conclusions:**

These observations suggest a critical role for the MyD88 pathway in initiating neuropathic pain, but a distinct role for the TRIF pathway and interferon in regulating neuropathic pain phenotypes in male mice.

## Background

Clinical symptoms arising from physical injury to the peripheral nerve include initiation of a pain behavior phenotype by otherwise innocuous stimuli (allodynia) [1]. Though complex, a number of components have been identified as associated with this pain phenotype. Such a behavioral phenotype has been identified in preclinical mononeuropathy models (e.g., spinal nerve ligation (SNL)), where a robust tactile allodynia [2-4] and evidence of an aversive state [5] have been noted. Based on these preclinical behavioral models, many contributors to the post-nerve injury pain state have been implicated, including changes in dorsal root ganglion (DRG), transcription factor expression, and activation of spinal glia [6-9], along with the appearance of inflammatory mediators such as tumor necrosis factor (TNF) [6,10-13]. Thus, after ligation of the L5 nerve, expression of activation transcription factor 3 (ATF3) in the DRG is increased [14,15]. In the spinal cord, microglia and astrocytes display morphological signs of activation in the ipsilateral lumbar enlargement of the spinal cord [16,17]. The reduction of injury-evoked hyperalgesia, with intrathecal (IT) inhibitors of glial activation, supports the contribution of glia as a possible target [18-22].

Toll-like receptors (TLRs) are a family of 14 identified receptors that play a key recognition role in the innate immune response. Several TLRs activate multiple downstream cascades that variously activate NF-κB dependent cytokine production and NF-κB independent interferon (IFN) induction [23,24]. These receptors are expressed on cells active in innate immunity, but are also found on glial (microglia and astrocytes) and non-glial (spinal and DRG neurons) CNS cells [25-27]. The role of peripheral and central glial activation in the neuropathic pain state raises the likelihood that TLRs may play a role in mediating spinal sensitization, initiated by peripheral nerve injury [28-30]. Current research indeed supports the importance of TLR signaling in neuropathic pain [31-36] and in persistent arthritic models where a neuropathic transition has been hypothesized [37-39]. In particular, TLR4 has been implicated as playing a critical role in the development of neuropathic pain in male rodents, but this may not be universally true in females [34,40-42]. While these studies indicate that TLRs play a role in these models, the cascades through which they mediate behaviorally defined pain states remain unclear.

**Figure 1:**
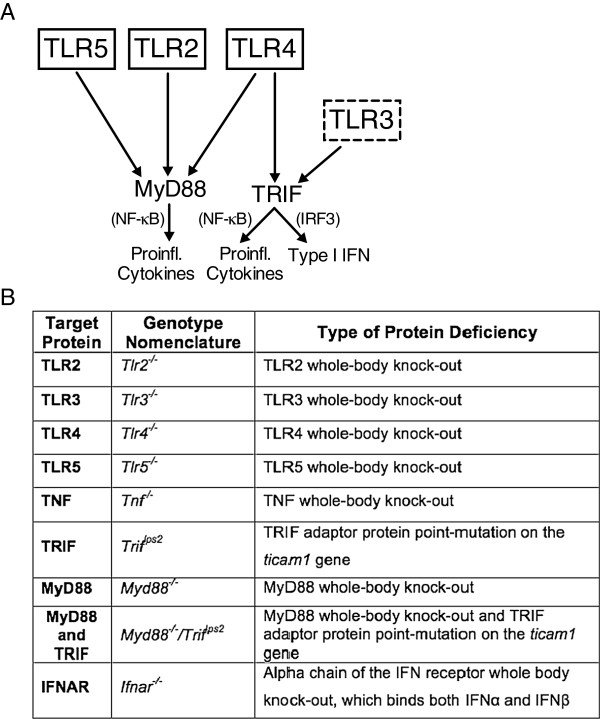
**Schematic of the TLR pathways. (A)** This figure highlights the key TLRs and their relevant pathways in this paper. TLR2, TLR4, and TLR5 are found on the cell surface, while TLR3 is in the cell endosomes. MyD88 is a key adaptor protein for all TLRs except TLR3. TLR3 and TLR4 signal through TRIF, resulting predominantly in type I interferon production. **(B)** This table summarizes the strains of mice used in the presented studies and the nomenclature used throughout the paper.

As indicated schematically in Figure 1A, TLRs signal through a limited number of adaptor proteins that converge in signaling through myeloid differentiation primary response gene 88 (MyD88) or TIR-domain-containing adapter-inducing IFNβ (TRIF). The MyD88 activation pathway, common to all TLRs except TLR3, leads to activation of NF-κB, producing pro-inflammatory cytokines such as TNF and IL-1β [43]. In contrast, the TRIF pathway is shared only by TLR3 and TLR4 signaling, and can result, by activation of IFN regulatory factors (IRF), in IFNβ secretion [44-46].

In a recent publication, we showed that a prominent tactile allodynia was produced by IT TLR agonists in male mice. These actions had several defining characteristics, namely i) spinal TLR2, 3, and 4 ligands initiate activation, though their respective receptors, and produce allodynia through TIRAP and TRIF signaling; ii) spinal TLR4 allodynia-inducing signaling is TNF dependent and TLR3 is TNF independent; and iii) unexpectedly, TRIF pathway activation repressed TLR4-induced allodynia, likely by an effect evoked by IFNβ production and signaling [47]. In the following study, we have extended this systematic assessment of the roles of individual spinal TLRs and the MyD88 and TRIF adaptor proteins in tactile allodynia and surrogate marker activation associated with the L5 SNL mononeuropathy model in male mice. The results herein, regarding the respective TLR-null male mice, show a strong correlation with the hypothesized role of spinal TLRs in initiating and regulating a persistent hyperalgesic state. Over the course of this work, it was observed that the nerve injury-induced tactile allodynia was comparable in male and female mice; however, given the recent report of a difference in TLR4 mediated spinal pain state [41], we also examined L5 SNL in *Tlr4*^*-/-*^ females.

## Methods

### Animals

All animal experiments were carried out according to protocols approved by the Institutional Animal Care and Use Committee of the University of California, San Diego (under the Guide for Care and Use of Laboratory Animals, National Institutes of Health publication 85-23, Bethesda, MD, USA). Mice were housed up to four per standard cage at room temperature and maintained on a 12-hour light/dark cycle (lights on at 07:00 AM). Testing was performed during the light cycle; food and water were available *ad libitum*. C57BL/6 mice (male, 25–30 g; female 20–25 g) were purchased from Harlan (Indianapolis, IN, USA). *Tlr2*^*-/-*^, *Tlr3*^*-/-*^, *Tlr4*^*-/-*^, and *Myd88*^*-/-*^ mice were a gift from Dr. S. Akira (Osaka University, Japan) [48-50] and were bred for 10 generations onto the C57BL/6 background. *Trif*^*lps2*^ mice were a gift from Dr. B. Beutler (UT Southwestern, TX, USA) and were directly generated on the C57BL/6 background [51]. The appropriate strains were intercrossed to generate *Myd88/Trif*^*lps2*^ mice. *Tlr5*^*-/-*^ and *Tnf*^*-/-*^ mice were purchased from The Jackson Laboratory. *Ifnar1*^*-/-*^ mice were originally obtained from B&K Universal Limited (Hull, UK) and backcrossed over 10 generations onto the C57BL/6 background. *Irf3*^*-/-*^ and *Irf7*^*-/-*^ were a gift from T. Taniguchi (University of Tokyo, Japan) [52,53]. Figure 1B lists the mouse genotypes and nomenclature used throughout the paper. Additional studies were performed with female C57BL/6 female *Tlr4*^*-/-*^ mice.

### L5 spinal nerve ligation model for neuropathic pain

Ligation of the L5 spinal nerve was performed as previously described [3]. Briefly, mice were anesthetized in a box of 2.5% vaporized isoflurane, with 2% oxygen and 2% room air, then placed in a nose cone for breathable anesthetic (2% isoflurane). The L5 spinal nerve root was surgically located, exposed and permanently ligated. The surgical opening was sutured and the mouse was closely monitored and allowed to recover in a heated cage until all reflexes were normal. Each mouse was assessed for any mobility impairments, and if none were present, then cleared for study. For the sham operation, the L5 spinal nerve is surgically exposed, but not ligated. Tactile threshold testing is carried out on days 7, 9, 12, and 14 post-surgery.

### Behavioral tests

Mechanical sensitivity was assessed using the von Frey up-down method. Filaments with values ranging from 2.44 g to 4.31 g (0.03 g to 2.00 g) were applied to the paw as previously described [54]. The 50% probability withdrawal threshold (in principal, the calculated force to which an animal reacts to 50% of the presentations) was recorded. The results from the ipsilateral (left) and contralateral (right) paws are graphed separately to show the unilateral tactile allodynia that results from the L5 SNL. Baseline tactile thresholds for all mouse strains used were compared via 1-way ANOVA and no significant differences were found in baseline thresholds.

### Intrathecal (IT) injection and drug delivery

The intrathecal needle placement procedure for the IT IFNβ (100 ng/5 μL) and IT vehicle (0.1% BSA) treatment was performed as previously described [36,55]. Briefly, mice were induced with 3% isoflurane (with 2% oxygen and 2% room air) in a chamber until a loss of the righting reflex was observed. A 1” 30-gauge needle attached to a 50 μL Hamilton syringe was inserted between the L5 and L6 vertebrae, evoking a tail flick reflex. Following recovery from anesthesia, as evidenced by a vigorous righting reflex and spontaneous ambulation, typically around 1–2 minutes, mice were evaluated for motor coordination and muscle tone. Tactile thresholds were measured using the up-down application of von Frey hairs along the following time course: 0 (baseline), 120-, and 240-minutes, and 24-h after IT treatment. IFNβ (Chemicon, 100 ng/5 μL in 0.1% BSA) was administered intrathecally as a post-treatment, 12 days after L5 SNL. In a separate group of mice, IFNβ (7,500 U/100 μL) or vehicle was administered i.p. on day 12. Tactile thresholds were measured using the up-down application of von Frey hairs along the following time course: 0 (baseline), 120-, and 240-minutes, and 24-h after i.p. treatment. In a third cohort of mice, IFNβ (7,500 U/100 μL) or vehicle was administered i.p. as a pre-treatment on day 0 (before L5 SNL), and days 2, 3, 4, 5, 6, and 7 after L5 SNL.

### Immunohistochemistry

Mice were deeply anesthetized with euthasol and perfused intracardially with 0.9% saline followed by 4% paraformaldehyde. The lumbar spinal cord and L4–L6 DRGs were removed, post-fixed in 4% paraformaldehyde over-night, and cryoprotected in 30% sucrose. Spinal cord sections (L4–L6) of the spinal cord were cut as free-floating sections (30 μm). Tissue sections were incubated with anti-GFAP antibody (1:20,000, Chemicon), and anti-Iba1 antibody (1:1,000, Wako). Binding sites were visualized with secondary antibodies conjugated with fluoro-Alexa-488 and Alexa-594 (1:500, Molecular Probes). Images were captured by Leica TCS SP5 confocal imaging system and quantified using ImageJ64 v.1.47 software. Glia reactivity is characterized by an increase in the number of cells and in the cell morphology (rounding of the cell bodies and thickening of processes) leading to an increase in labeling with increasing glia reactivity. Staining for microglia (Iba1) and astrocyte (GFAP) were each quantified by measuring the total integrated intensity area of pixels divided by the total number of pixels in a standardized area of the dorsal horn (background). The investigator was blinded to experimental conditions during the quantification. Staining intensity was examined in laminae I–III of the superficial dorsal horn with four to five sections (separated by at least 180 μm) examined per animal and three to four animals per experimental condition. Only the areas above a background pixel intensity threshold were included. An increase in the integrated intensity/pixel area for Iba1 and GFAP staining was interpreted to signify microglia and astrocyte reactivity, respectively. All data are presented as fold change from the background sample. Each mouse had its own background sample taken per tissue section from the deeper lamina to control for staining variation. Background values were initially divided into ipsilateral and contralateral halves and assessed for staining intensity as described above. The ipsilateral value was divided by the contralateral value to ensure that the background sample was consistent with an ipsi/contra value of 1. Iba-1 background values were 1.04 ±0.04 and GFAP values 0.98 ±0.08, confirming an even background staining. Statistics were performed on raw data values.

ATF3 staining was assessed by counting the number of cells with ATF3 positive nuclei. The L5 ipsilateral and contralateral DRGs were cut in transverse sections (10 μm) and mounted on glass slides. DRGs were incubated with anti-ATF3 (1:1,000, Santa Cruz) and DAPI. Binding sites were visualized with secondary antibodies conjugated with fluoro-Alexa-488. All quantified sections were separated by at least 60 μm. ATF3 positive nuclei were counted (one section per animal) and three to four animals are included for each group.

### Statistics

Data are presented as group mean ± SEM. Tactile threshold time course curves (plotted as the mean ± SEM vs. time after treatment) were analyzed with a one-way analysis of variance (ANOVA) with repeated measures over time, followed by Dunnett’s *post hoc* test to compare each time point to the same group’s baseline. Second, to compare behavior between the two mouse strains, a 2-way ANOVA and Bonferroni *post hoc* test was used comparing mouse group and treatment. For staining intensity data was compared across mouse strains and ipsilateral vs. contralateral with a 2-way ANOVA followed by Bonferroni *post hoc* test. All analyses employed Prism statistical software, CA, USA.

## Results

### Contributions of TLR signaling to allodynia in spinal nerve ligation

**Figure 2:**
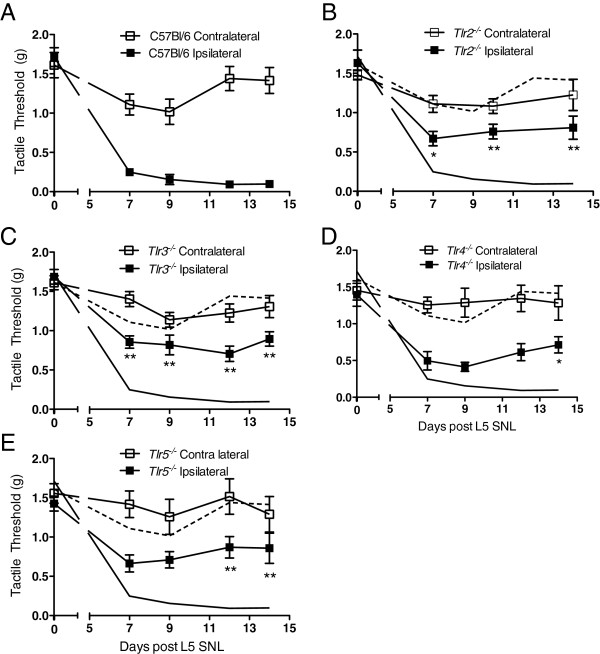
**Unilateral tactile allodynia observed following L5 SNL in C57BL/6 mice reduced in TLR signaling deficient mice.** L5 SNL was performed on **(A)** C57BL/6, **(B)***Tlr2*^*-/-*^, **(C)***Tlr3*^*-/-*^, **(D)***Tlr4*^*-/-*^, and **(E)***Tlr5*^*-/-*^ mice. Mice were allowed to recover and were tested at days 7, 9, 12 and 14 post-SNL. The solid black line and dashed line represent the C57BL/6 ipsilateral and contralateral thresholds, respectively, on **B-E**. **(A)** C57BL/6 mice show a robust tactile allodynia in the ipsilateral paw beginning 7-days post-surgery. The **(B)***Tlr2*^*-/-*^, **(C)***Tlr3*^*-/-*^, **(D)***Tlr4*^*-/-*^, and **(E)***Tlr5*^*-/-*^ all produced a reduction in the ipsilateral paw tactile threshold following L5 SNL, but none completely reversed nerve injury-induced allodynia. Data are expressed as mean ± SEM (n = 5–8 mice/group) and analyzed via 2-way ANOVA, followed by Bonferroni *post hoc* test to compare each time point to the respective WT C57BL/6 group, ipsilateral or contralateral (*P <0.05 or **P <0.01).

Unilateral L5 SNL model produces a robust ipsilateral reduction (i.e., from 1.7 g to <0.2 g in the ipsilateral paw (Figure 2A), versus 1.7 g to 1–1.5 g in the contralateral paw). Similar results were observed in the wild type (WT) female mice in the tactile stimulus required to initiate a withdrawal of the stimulated paw (Additional file 1: Figure S1). In *Tlr2*^*-/-*^, *Tlr3*^*-/-*^, and *Tlr5*^*-/-*^ mice, there was no change in pre-surgery tactile baselines as compared to WT mice (Figure 2); however, in such mice, there was a persistent and significant reduction in tactile allodynia (Figure 2B-E). *Tlr4*^*-/-*^ mice had more severe allodynia on days 7 and 9 and then recovered to the 50% level on days 12 and 14 (Figure 2E). In contrast to the male *Tlr4*^*-/-*^ mice, the female *Tlr4*^*-/-*^ mice did not show a reduction in the observed tactile allodynia (Additional file 1: Figure S1).

**Figure 3:**
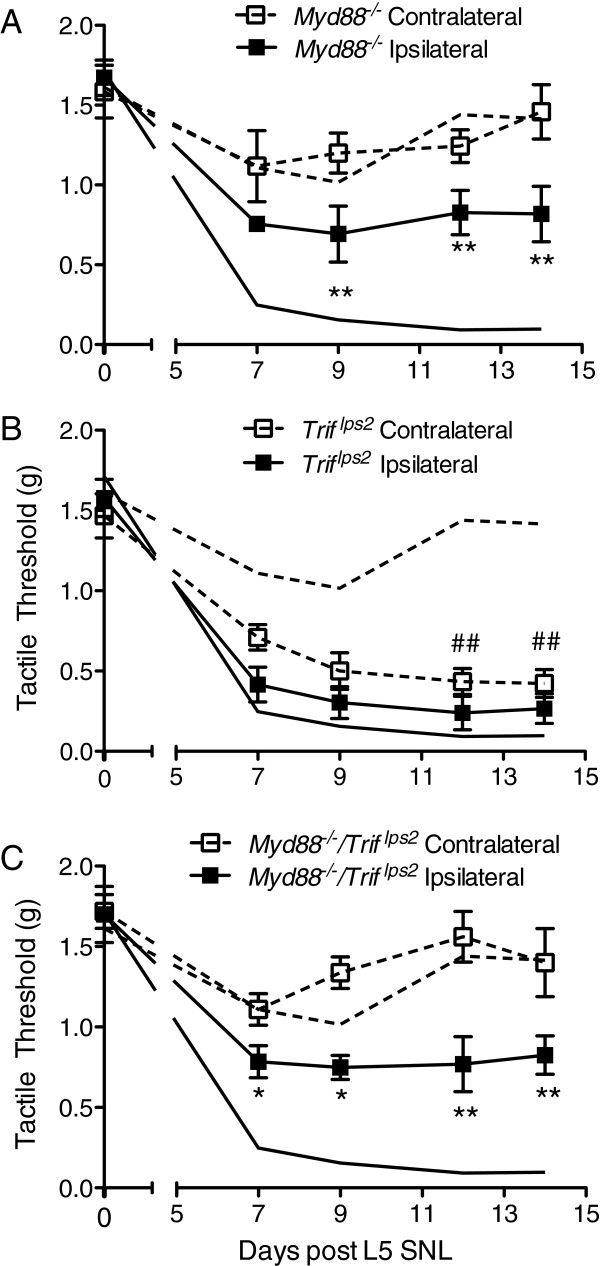
**TRIF is important for unilateral tactile allodynia phenotype following L5 SNL.** L5 SNL was performed on **(A)***Myd88*^*-/-*^, **(B)***Trif*^*lps2*^, and **(C)***Myd88*^*-/-*^*/Trif*^*lps2*^ mice. Mice were allowed to recover and were tested at days 7, 9, 12, and 14 post-SNL. The solid black line and dashed line represents the C57BL/6 ipsilateral and contralateral thresholds, respectively. Both **(A)***Myd88*^*-/-*^ and **(C)***Myd88*^*-/-*^*/Trif*^*lps2*^ ipsilateral tactile thresholds were significantly different from the C57BL/6 thresholds beginning 7 days post-L5 SNL. Surprisingly, the **(B)***Trif*^*lps2*^ mice displayed a tactile allodynia in both ipsilateral and contralateral paws. The *Trif*^*lps2*^ ipsilateral paw thresholds were not significantly different from the C57BL/6 mice, but the contralateral paws were significantly different beginning at day 12 post-L5 SNL. Data are expressed as mean ± SEM (n = 5–8 mice/group) and analyzed via 2-way ANOVA, followed by Bonferroni *post hoc* test to compare each time point to the respective C57BL/6 group, ipsilateral or contralateral (*P <0.05 or **P <0.01 for ipsilateral group; ##*P* <0.01 for contralateral group).

TLR2 and TLR5 signal through MyD88, and TLR3 signals through TRIF. TLR4 utilizes both the MyD88 and TRIF pathways (Figure 1A). Both *Myd88*^*-/-*^*/Trif*^*lps2*^ and *Myd88*^*-/-*^ mice after SNL had an ipsilateral paw threshold that was significantly higher than C57BL/6 mice (Figure 3A,C). Surprisingly, mice deficient in TRIF signaling produced a robust tactile allodynia in both the ipsilateral and contralateral paws following SNL (Figure 3B). This suggests that the TRIF pathway is involved in the absence of bilateral effects produced by an otherwise unilateral mononeuropathy. Since the *Myd88*^*-/-*^*/Trif*^*lps2*^ mice only developed ipsilateral pain-like behavior, MyD88 is required for contralateral allodynia to develop (Figure 3C).

Since these mice are deficient in immune response signaling, we wanted to ascertain that surgery itself was not initiating the observed hypersensitivity. C57BL/6 and *Trif*^*lps2*^ mice underwent a sham SNL procedure, similar in all respects to the protocol followed for the SNL, except that the L5 spinal nerve was surgically exposed, but not ligated. This resulted in no significant change in tactile thresholds following the procedure or any difference between the two mouse strains (Additional file 2: Figure S2).

### Dorsal horn glial activation

**Figure 4:**
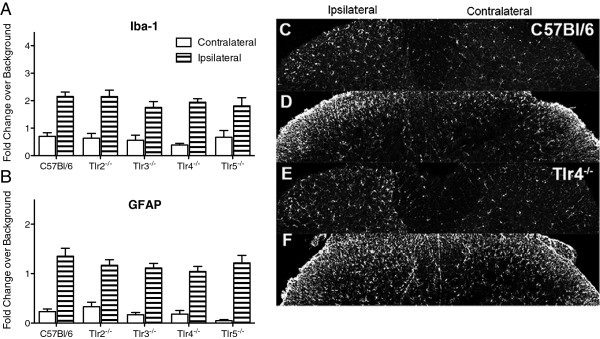
**Glial activation in lumbar spinal cord following L5 SNL.** At day 14 following L5 SNL, the lumbar region of the spinal cord was harvested and incubated with antibodies against Iba-1 and GFAP. Both **(A)** Iba-1 and **(B)** GFAP immunoreactivity is consistently elevated in the ipsilateral side of the dorsal horn compared to contralateral within each group. Data are expressed as mean ± SEM (n = 3–5 sections per mouse, with 3–4 mice/group) and analyzed via 2-way ANOVA followed by Bonferroni *post hoc* test. No differences were found across mouse strains. Representative stained images are presented from C57BL/6 mice with **(C)** Iba-1 and **(D)** GFAP staining and from *Tlr4*^*-/-*^ mice with **(E)** Iba-1 and **(F)** GFAP staining.

**Figure 5:**
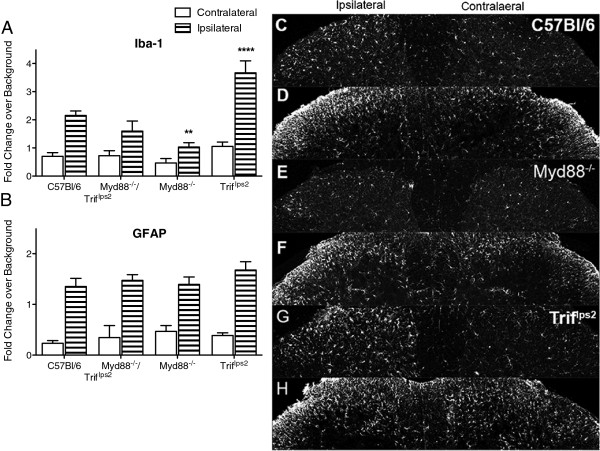
**TRIF and MyD88 signaling deficient mice have different Iba-1 immunoreactivity profiles following L5 SNL.** At day 14 following L5 SNL the lumbar region of the spinal cord was harvested and incubated with antibodies against Iba-1 and GFAP. Both **(A)** Iba-1 and **(B)** GFAP immunoreactivity were consistently significantly elevated in the ipsilateral side of the dorsal horn compared to contralateral within each group. Data expressed as mean ± SEM (n = 3–5 sections per mouse, with 3–4 mice/group) and analyzed via 2-way ANOVA followed by Bonferroni *post hoc* test. The Iba-1 *Trif*^*lps2*^ and *Myd88*^*-/-*^ ipsilateral groups are statistically different from the C57BL/6 ipsilateral group (**P <0.01; ****P <0.0001). No significant difference was found among the contralateral groups. Representative stained images are presented from C57BL/6 mice with **(C)** Iba-1 and **(D)** GFAP, *Myd88*^*-/-*^ mice with **(E)** Iba-1 and **(F)** GFAP, and *Trif*^*lps2*^ mice with **(G)** Iba-1 and **(H)** GFAP, which support the quantified immunoreactivity results.

Spinal Iba-1 and GFAP have previously been shown to be upregulated in the lumbar spinal cord ipsilateral to the nerve injury in C57BL/6 mice. Lumbar spinal tissue was collected at 14 days post-L5 SNL and incubated with antibodies for both Iba-1 and GFAP. Quantification of both Iba-1 and GFAP show distinct increases in immunoreactivity ipsilateral to the side of injury in laminae I–III at this time point (Figure 4A,B). There was no significant difference among the C57BL/6 animals and any of the specific TLR deficient animals; representative stained images show this ipsilateral increase (Figure 4C–F, Additional file 3: Figure S3). The *Myd88*^*-/-*^ mice showed less ipsilateral Iba-1 activation as compared to the C57BL/6 ipsilateral activation, while there was no difference in GFAP (Figure 5A,B,E,F). The *Trif*^*lps2*^ mice, which developed both ipsilateral and contralateral tactile allodynia, showed a significant increase in the ipsilateral Iba-1 activation and a numerical increase in the contralateral Iba-1 immunoreactivity that did not reach statistical significance and no difference in GFAP on either side (Figure 5A,B,G,H). Representative staining images, which were used for the Iba-1 and GFAP quantification, are shown in Additional file 3: Figure S3.

### Dorsal root ganglia ATF3 expression

**Figure 6:**
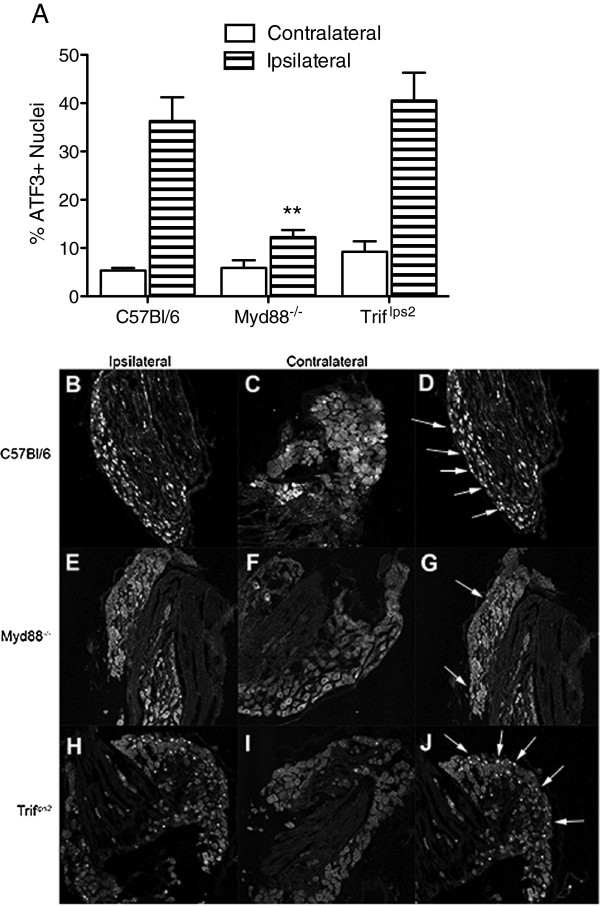
**MyD88 signaling deficient mice have less ATF3+ nuclei following L5 SNL.** At day 14 following L5 SNL, the ipsilateral and contralateral L5 DRGs were harvested and incubated with an antibody against ATF3. **(A)***Myd88*^*-/-*^ DRGs display less ATF3 immunoreactivity in their L5 ipsilateral DRGs when compared to C57BL/6. No significant difference was found among the other groups. Data expressed as mean ± SEM (n = 4–8 mice/group) and analyzed via 2-way ANOVA followed by Bonferroni *post hoc* test. The *Myd88*^*-/-*^ ipsilateral group is statistically different from the C57BL/6 ipsilateral group (**P <0.01). Representative stained images from the DRGs of C57BL/6 **(B-D)**, *Myd88*^*-/-*^**(E-G)**, and *Trif*^*lps2*^**(H-J)** mice are presented. Examples of ATF3+ nuclei are marked with a white arrow in **D**, **G**, and **J**.

To assess the afferent response to nerve injury following SNL, DRGs were incubated with antibodies for ATF3. In the absence of injury, very few C57BL/6 DRGs showed ATF3 expression. However, following surgery, approximately 40% of the ipsilateral L5 DRG neurons displayed ATF3 positive nuclei (Figure 6A). In the DRGs from *Myd88*^*-/-*^ mice, the percent of ATF3-positive DRG neurons fell to around 20% suggesting that inhibition of MyD88, or all TLR signaling, except TLR3, diminished the consequence of the SNL. The signaling mutation in TRIF did not affect the number of ATF3 positive nuclei (Figure 6A). In the TRIF deficient animals there was also no increase of ATF3 positive nuclei in the contralateral DRGs. Representative stained images are presented with white arrows labeling examples of ATF3 positive nuclei (Figure 6B-J; Additional file 4: Figure S4).

### Type I interferon mediates TRIF regulation of contralateral allodynia

**Figure 7:**
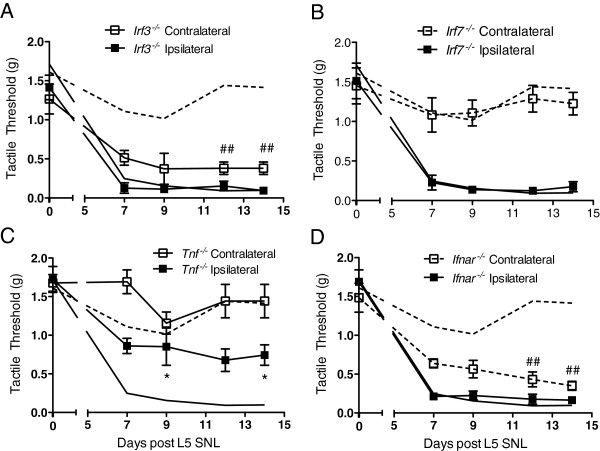
**IRF3 and IFNR signaling is required for regulation of contralateral tactile allodynia following L5 SNL.** L5 SNL was performed on **(A)***Irf3*^*-/-*^, and **(B)***Irf7*^*-/-*^, **(C)***Tnf*^*-/-*^, and **(D)***Ifnar*^*-/-*^ mice. Mice were allowed to recover and were tested at days 7, 9, 12, and 14 post-SNL. The solid black line and the dashed line represent the C57BL/6 ipsilateral and contralateral thresholds, respectively. The *Irf3*^*-/-*^**(A)** and *Ifnar*^*-/-*^**(D)** mice displayed a tactile allodynia in both the ipsilateral and contralateral paws. Conversely, the *Irf7*^*-/-*^**(B)** and *Tnf*^*-/-*^**(C)** mice only produced a drop in the ipsilateral paw threshold. There was no statistical significance between the C57BL/6 mice and these mice. Data expressed as mean ± SEM (n = 5–8 mice/group) and analyzed via 2-way ANOVA, followed by Bonferroni *post hoc* test to compare each time point to the respective C57BL/6 group, ipsilateral or contralateral (*P <0.05 or **P <0.01 for ipsilateral group; ##*P* <0.01 for contralateral group).

To further delineate the pathway responsible for the contralateral drop in thresholds in the *Trif*^*lps2*^ mice, we compared two strains deficient in IFN regulatory factors (IRF) that are known TLR signaling regulators. IRF3 is downstream of TRIF (TLR3 and TLR4), and IRF3 signaling deficient mice (*Irf3*^*-/-*^) have the same contralateral and ipsilateral allodynia profile following L5 SNL (Figure 7A). This confirmed the effect of the *Trif*^*lps2*^ mice previously observed (Figure 3B). TLR7 and TLR9 signaling through MyD88 and IRF7 can also produce type I IFNs, primarily IFNα. In *Irf7*^*-/-*^ mice following L5 SNL, a robust ipsilateral tactile allodynia was observed and the basal contralateral paw threshold was maintained, just as in the C57BL/6 and *Myd88*^*-/-*^ mice (Figure 3A and Figure 7B).

These results indicated that the expression of the contralateral pain phenotype after unilateral nerve injury was regulated by the TRIF-IRF3 pathway. Stimulation of TLRs through MyD88 and TRIF can result in proinflammatory cytokines such as TNF through NF-κB or type I IFNs via IRF3, respectively. Thus, we looked at the L5 SNL model in mice deficient in TNF and type 1 IFN receptors (Figure 7C and D). These *Tnf*^*-/-*^ and *Ifnar*^*-/-*^ mice exhibited the same tactile allodynia profile as the mice deficient in MyD88 and TRIF signaling, respectively.

**Figure 8:**
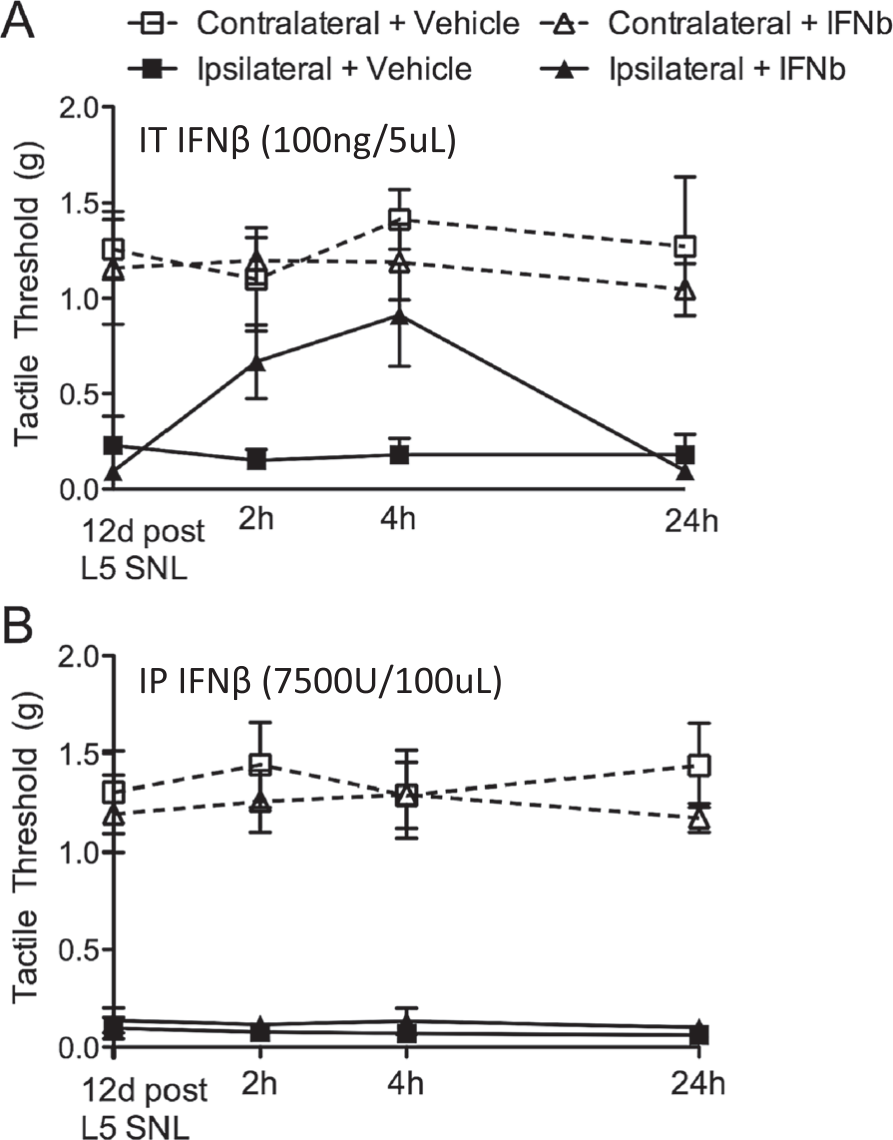
**IT IFNβ transiently reverses L5 SNL induced tactile allodynia.** Twelve days following L5 SNL, C57BL/6 mice received IFNβ either **(A)** intrathecally or **(B)** intraperitoneally. **(A)** IT IFNβ (100 ng/5 μL) transiently reversed the tactile allodynia present at 12 days post-L5 SNL in C57BL/6 mice. **(B)** IFNβ i.p. (7,500 U/100 μL) did not affect the tactile allodynia present at 12 days post-L5 SNL in C57BL/6 mice. Data expressed as mean ± SEM (n = 3–4 mice/group) and analyzed via 2-way ANOVA. Following IFNβ treatment there was a significant effect of the IT treatment **(A)** (*P* <0.05), but not the i.p. treatment **(B)**.

Since *Trif*^*lps2*^, *Irf3*^*-/-*^, and *Ifnar*^*-/-*^ mice displayed both ipsilateral and contralateral allodynia following SNL, we further hypothesized that IFNβ could be a regulatory mediator in the nociceptive processing initiated by nerve injury. C57BL/6 mice treated with IFNβ intrathecally (100 ng/5 μL) at day 12 post-L5 SNL produced a transient reversal (lasting longer than 4 hours but less than 24 hours) in the L5-SNL-induced tactile allodynia (Figure 8A). When IFNβ was administered i.p. (7,500 U/100 μL), no effect on tactile thresholds was observed (Figure 8B). In other models, IFNβ is often administered not in a single dose, but as a multi-dose pretreatment. Thus, we gave C57BL/6 mice six doses of IFNβ i.p. (7500 U/100 μL) beginning at day 0, before L5 SNL, and continuing daily through day 7. This prolonged IFNβ i.p. treatment regimen also had no effect on tactile thresholds (Additional file 5: Figure S5).

## Discussion

This study examines the role of TLRs and their respective adaptor proteins on tactile allodynia associated with L5 SNL mononeuropathy. There are several pivotal observations:

i) The robust unilateral tactile allodynia initiated by L5 SNL was incompletely reversed in MyD88, or MyD88/TRIF signaling-deficient male mice. These mice displayed only a partial reduction in withdrawal thresholds and slightly reduced ipsilateral Iba-1 immuno-reactivity. This indicates that the partial reduction in tactile allodynia, as compared to C57BL/6 mice, reflects events other than those mediated by TLRs alone. This is consistent with the observation that *Tlr2*^*-/-*^, *Tlr3*^*-/-*^, *Tlr4*^*-/-*^, and *Tlr5*^*-/-*^ mice showed a comparable partial reduction in tactile allodynia, as compared to C57BL/6 mice.

ii) *Tnf*^*-/-*^ male mice showed only a partial reduction in the L5 SNL-induced tactile allodynia indicating alternate/additional downstream cytokine mediators for tactile allodynia following L5 SNL.

iii) *Trif*^*lps2*^ male mice developed profound ipsilateral and contralateral tactile allodynia, suggesting an enhanced response to the L5 SNL. Disruption of MyD88 prevented contralateral tactile allodynia in the *Myd88*^*-/-*^*/Trif*^*lps2*^ male mice, suggesting a critical role for the MyD88 pathway in initiating neuropathic pain.

iv) IFNβ administered intrathecally transiently reversed the L5 SNL, suggesting a distinct role for the TRIF/IRF3 pathway and interferons in regulating the expression of the neuropathic pain phenotype.

v) In contrast to the male *Tlr4*^*-/-*^ mice, the female *Tlr4*^*-/-*^ mice did not show a reduction in the observed tactile allodynia following L5 SNL.

**Figure 9:**
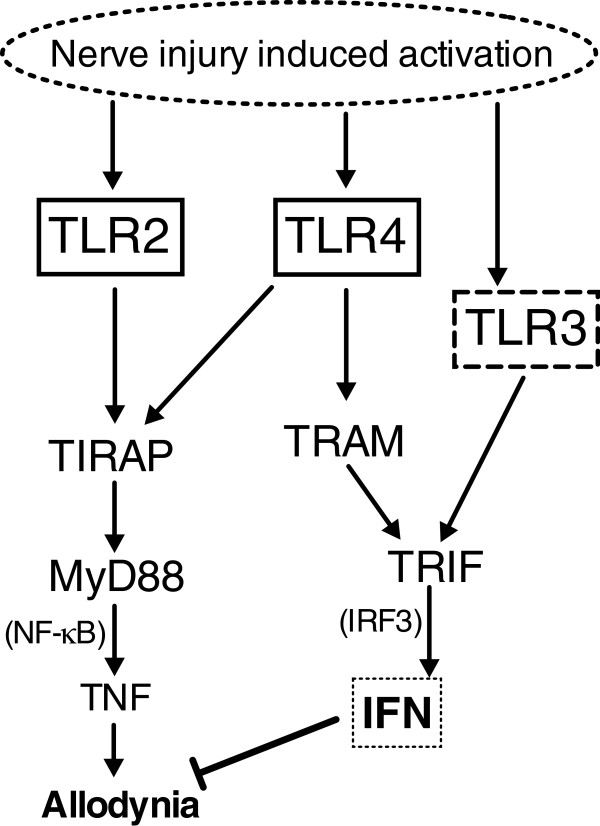
**Hypothesis for the development of tactile allodynia following nerve injury.** TLR 2 and 4 are expressed on the surface membrane (solid outline), signal through the adapters TIRAP and MyD88 resulting NF-κB activation and proinflammatory cytokine expression such as TNF. Alternatively, TLR4 can signal through adaptors TRAM and TRIF. TLR3 is expressed in the endosome (dotted outline) and signals through TRIF. TRIF can activate IRF3 and initiate IFNβ transcription. We hypothesize that there is an initial endogenous TLR activation signal following nerve injury that results in: i) concurrent activation of multiple TLRs; ii) the TLR2 and TLR4 TIRAP-MyD88 pathway and production of TNF produces an ipsilateral and contralateral tactile allodynia; and iii) the TLR3-TRIF pathway suppresses contralateral allodynia development associated with interferon release (dotted square) and downstream interferon receptor signaling.

The partial effects of L5 SNL on tactile allodynia in *Tlr2*^*-/-*^ and *Tlr4*^*-/-*^ male mice are comparable to previous reports [31,56]. The lack of TNF signaling diminished, but did not prevent, L5 SNL induced nociception, a finding also comparable to other reports where TNF ablation alone was insufficient to completely inhibit nerve injury-induced allodynia [57]. These results, however, show that MyD88 signaling is required for the development of nerve injury evoked tactile allodynia. Conversely, TRIF is important not only for recovery following nerve injury-induced allodynia, but also in the absence of TRIF signaling a unilateral nerve injury yields an unexpected bilateral effect. This TRIF action in developing neuropathy is mediated at least in part through IFNβ (Figure 9), suggesting an intrinsic regulatory action upon nerve injury-induced changes in nociceptive processing.

### Spinal TLR signaling and allodynia

As noted above, we have previously characterized downstream signaling and behavioral effects arising from the activation of spinal TLR2, 3, or 4 [47]. IT delivery of the respective agonists evoked a robust and persistent tactile allodynia by a specific action on their respective TLRs. Consistent with their downstream signaling cascade (Figure 1A), in TIRAP signaling-deficient mice (downstream to TLR2 and TLR4), allodynia after IT TLR2 ligand and TLR4 ligand was abolished. Unexpectedly, however, in TRIF (*Trif*^*lps2*^) signaling-deficient mice (downstream of TLR3 and TLR4), TLR3 mediated tactile allodynia was abolished, but spinal TLR4 activation produced an unexpectedly persistent and robust enhancement (>21 days) in tactile allodynia. Consistent with a role of IFNβ (downstream to TRIF) in regulating recovery after IT TLR4 ligand, a persistent tactile allodynia was also noted in IFN signaling-deficient mice. Additionally, through the use of *Tnf*^*-/-*^ mice, we noted that spinal TIRAP and TRIF cascades differentially lead to robust tactile allodynia by TNF-dependent and -independent pathways, respectively.

We believe that these previous studies provide a mechanistic framework for interpreting the results observed in the SNL mononeuropathy-mediated tactile allodynia. Thus, we emphasize the following: i) disruption of TRIF/IFN signaling leads to an enhanced and prolonged spinal response, suggesting that the TRIF pathway is involved in the recovery following nerve injury; ii) disruption of MyD88 signaling blocked the effects of the respective TLRs, completely preventing contralateral tactile allodynia in the *Myd88*^*-/-*^*/Trif*^*lps2*^ mice; thus, MyD88 is required for contralateral tactile allodynia to develop; and, iii) IT IFNβ indeed produced a transient reversal of the observed L5 SNL-induced tactile allodynia.

### Contralateral allodynia and unilateral nerve injury

The development of a bilateral effect as a result of MyD88 signaling in the male *Trif*^*lps2*^ mice is of particular interest. While the common mononeuropathy pain phenotype in humans and in experimental animal models is an ipsilateral change in sensitivity and dysesthesia, contralateral (bilateral) effects have been described. Such bilateral expression has been noted in the earliest clinical reports [58-65]. In preclinical models, examples of peripheral mononeuropathy producing contralateral effects are primarily limited to the homotopic contralateral segments [58,66,67]. Thus, chronic unilateral compression of the L4 and L5 DRGs in mice produced a strong mechanical and thermal hyperalgesia, as well as tactile allodynia in the ipsilateral paw, which has been reported to spread to the contralateral regions along the same time course [68]. In a rat model of chronic post-cast pain, two-week immobilization due to casting resulted in induced atrophy and inflammatory changes and widespread hyperalgesia that lasted 5 to 10 weeks after cast removal [69].

The mechanism of this onset of bilaterality is not known. As noted, in these studies, the homolaterality excludes a systemic humoral signal. In a model of unilateral intraplantar capsaicin, the c-fos activation pattern of spinal glycineric and GABAergic neurons increased on the opposite spinal relative to the injection [70]. Following unilateral nerve ligation there was a long lasting loss of PGP9.5^+^ cutaneous innervation contralaterally to the unilateral nerve injury, suggesting that transcellular signals link homologous neurons on opposite sides of the body [58]. Alternatively, such bilateral actions may reflect the aberrant activation of the bulbospinal facilitator pathways [71,72]. In the present study, the absence of an increase in ATF3, however, suggests that there was no bilateral effect on afferent nerve integrity. The present studies offer an important potential underlying mechanism. Here, we show that *Trif*^*lps2*^ mice developed strong contralateral allodynia while *Myd88*^*-/-*^*/Trif*^*lps2*^ mice only developed ipsilateral allodynia, indicating that MyD88 is required for contralateral allodynia to develop. These results offer the provocative hypothesis that following unilateral nerve injury a bilateral effect mediated by several mechanisms may be the normal occurrence, in the absence of a TRIF-IFN dependent inhibition.

Since both *Trif*^*lps2*^ and *Ifnar*^*-/-*^ male mice produced both ipsilateral and contralateral allodynia, we hypothesize that type I IFN could play a major role in endogenous pain management following nerve injury. Other reports support this hypothesis since IFNβ induces MHC class I leading to enhanced axonal growth and motor function recovery following nerve injury [73]. C57BL/6 mice were treated with IT IFNβ at day 12 post-L5 SNL, which transiently reversed tactile allodynia. However, here, i.p. IFNβ treatment in both single dose and repetitive doses over 6 days early in the time course, did not improve the tactile thresholds.

### Role for endogenous ligands in nerve injury-induced allodynia

These studies display the complex cascades activated by nerve injury and reveal the likely role of endogenous ligands for the TLRs, which would be present in the DRG and spinal cord secondary to signaling generated by an injury of the peripheral axon in male mice. Products potentially released by neuraxial cells include high mobility group box 1 (HMGB1), tenacin C, peroxiredoxin (Prx) family proteins, β-amyloid (Aβ), hyaluronan, DNA or RNA immune complexes, heat shock proteins, and heparan sulfate [74-83]. An intriguing hypothesis is that, in addition to spinal glia and neurons, non-neuronal cells such as macrophages, which migrate into the spinal cord and DRG after peripheral nerve injury, could serve as one source of such neuraxial TLR activation [7].

An interesting observation was that after L5 SNL in C57BL/6 mice, the ipsilateral L5 DRG shows nearly 40% ATF3 positive nuclei. The DRG from *Myd88*^*-/-*^ mice showed only around 20% of ATF3 positive nuclei, suggesting that inhibition of MyD88, results in less DRG reactivity to the nerve injury. TNF, TRIF, or IFNR knockout alone did not affect the number of ATF3 positive nuclei. In terms of nerve injury recovery, the TRIF protein has been implicated in the activation of microglia and p38 MAPK to clear axonal debris following peripheral insult [84,85]. This would suggest that without the TRIF protein there would be more axonal debris present with the ability to activate local TLRs, which detect endogenous materials. More specifically, products such as HMGB1, Prx proteins, and other damage-associated molecular pattern molecules previously mentioned, can stimulate TLR2 and TLR4 [80,86]. It is important to emphasize that while the TLRs play a defined role in the inflammatory and injury-initiated components of the classical innate immune response, the research outlined here and elsewhere emphasizes that these TLR cascades play a role in the normal processing of sensory information generated by distant injury and inflammation.

### Sex

In the present work, the primary model examined the role of TLR receptors and adaptor proteins in the male mouse. As reviewed above, an evident role for the TLR cascade in this nerve injury induced allodynia was noted. Sorge et al. [41] reported that in female mice, knockout of TLR4 had no effect on mononeuropathic allodynia. In the present work, female *Tlr4*^*-/-*^ mice also continued to display a robust tactile allodynia following nerve injury. Accordingly, these results are in agreement with those reported previously. Additional studies will be required to consider the role of other TLR cascades in the post-nerve injury pain state and whether or not this effect is observed in models of both poly- and mono-neuropathy. Additionally, Sorge et al. [41] reported that spinal TLR4 binding sites were unaltered. This finding suggests several possible alternatives, including the lack of release of endogenous TLR4 agonists. However, the fact that Sorge et al. [41] reported that IT Lipopolysaccharide (LPS) had no pro-allodynic effect in the female mice (in contrast to the male) argues against the simple explanation that endogenous agents initiating a hyperpathic state through the TLR4 after nerve ligation were absent in the female. Future work addressing these sex differences for other TLR components will be of considerable interest. Until that time, it is necessary to explicitly note that the present results (and that of others in the area) apply to the male sex, until otherwise shown.

## Conclusions

The present studies uncovered three important roles of the TLR pathway in mononeuropathy in the male mouse: i) individual TLRs only modestly contribute to the allodynia present after nerve injury; ii) TRIF plays a dual role mediating allodynia arising from TLR3 activation and regulating, through an IFNβ pathway, the recovery following nerve injury-induced allodynia; and iii) the MyD88 pathway is required for the development of contralateral allodynia. Characterization of the agents activating these TLRs and the parameters governing their release will be of particular interest in defining the role of TLRs in nociceptive processing as illustrated by these studies. The roles of these cascades in the female mouse remain to be determined.

## Abbreviations

ATF3: Activating transcription factor 3; Aβ: β-amyloid; DAMP: Damage-associated molecular pattern molecules; DRG: Dorsal root ganglia; HMGB1: High mobility group box 1; IFN: Interferon; IL: Interleukin; IRF: Interferon regulatory factor; IT: Intrathecal; LPS: Lipopolysaccharide; MyD88: Myeloid differentiation primary response gene (88); Prx: Peroxiredoxin; SNL: Spinal nerve ligation; TIRAP: Toll-interleukin 1 receptor (TIR) domain containing adaptor protein; TRIF-TIR: Domain-containing adapter-inducing interferon-β; TLR: Toll-like receptor; TNF: Tumor necrosis factor.

## Competing interests

The authors are employees of the University of California, San Diego, and the work was supported by the NIH grants listed. The authors have no other relevant affiliations or financial involvement with any organization or entity with a financial interest in or financial conflict with the subject matter or materials discussed in the manuscript. This includes employment, consultancies, honoraria, stock ownership or options, expert testimony, grants or patents received or pending, or royalties.

## Authors’ contributions

JS and KE performed the SNL and animal testing. JS performed the immunohistochemistry, quantification, and statistics. MC bred and genotyped the mice. JC assisted in the quantification of the immunohistochemistry. JS, MC, and TY conceived of the study, participated in its design and coordination, and helped to draft the manuscript. All authors read and approved the final manuscript.

## Supplementary Material

Additional file 1: Figure S1 Unilateral TA observed following L5 SNL in C57BL/6 female mice was not reduced in TLR4 signaling-deficient female mice. L5 SNL was performed on female **(A)** C57BL/6 and **(B)***Tlr4*^*-/-*^ mice. Mice were allowed to recover and were tested at days 7, 9, 12, and 14 post-SNL. The solid black line and dashed line represent the C57BL/6 ipsilateral and contralateral thresholds. **(A)** C57BL/6 mice showed a robust TA in the ipsilateral paw beginning 7 days post-surgery. The **(B)***Tlr4*^*-/-*^ mice showed no effect upon the ipsilateral paw tactile threshold following L5 SNL. Data are expressed as mean ± SEM (n = 5 mice/group) and analyzed via 2-way ANOVA, followed by Bonferroni *post hoc* test to compare each time point to the respective WT C57BL/6 group, ipsilateral or contralateral (###*P* <0.01 vs. contralateral paw; ***post vs. baseline). Click here for file

Additional file 2: Figure S2 L5 SNL Sham produced no significant effect on tactile thresholds. C57BL/6 and *Trif*^*lps2*^ mice underwent L5 SNL sham surgery and tactile thresholds were measured. There were no significant differences between the tactile thresholds of the four groups as assessed by 1-way ANOVA. Click here for file

Additional file 3: Figure S3 Iba-1 and GFAP immunoreactivity following L5 SNL. At day 14 following L5 SNL, the lumbar region of the spinal cord was harvested and incubated with antibodies against Iba-1 and GFAP. Iba-1 immunoreactivity is visualized with Alexa-488 (green) in the left panel and GFAP with Alexa-594 (red) in the right panel. Quantification for the Iba-1 and GFAP immuno-reactivity is found in Figure 3 and Figure 5. Click here for file

Additional file 4: Figure S4 ATF3 immuno-reactivity following L5 SNL. At day 14 following L5 SNL, the L5 right and left DRGs were harvested and incubated with an antibody against ATF3. ATF3 immuno-reactivity is visualized with Alexa-488 (green) and recognized as an intense fluorescent mark in the nuclei region. The white arrows point to examples of ATF3 staining. Quantification for the ATF3 immuno-reactivity is found in Figure 6. Click here for file

Additional file 5: Figure S5 Repetitive treatment with IP IFNβ had no effect on tactile thresholds following L5 SNL. C57BL/6 mice received i.p. IFNβ (7,500 U/100 μL) or vehicle (0.1% BSA) as a pre-treatment on day 0 (before L5 SNL) and days 2, 3, 4, 5, and 6 after L5 SNL. Tactile thresholds were measured on days 7, 9, 12, and 14. No significant differences were found between the i.p. vehicle and i.p. IFNβ groups as assessed by 2-way ANOVA followed by Bonferroni *post-hoc* test. Click here for file
