# Supplementary figures and images for: Toll-like receptor signaling adapter proteins govern spread of neuropathic pain and recovery following nerve injury in male mice
Source: J Neuroinflammation. 2013 Dec 9;10:148. doi: 10.1186/1742-2094-10-148 (PMC3896749; doi:10.1186/1742-2094-10-148)

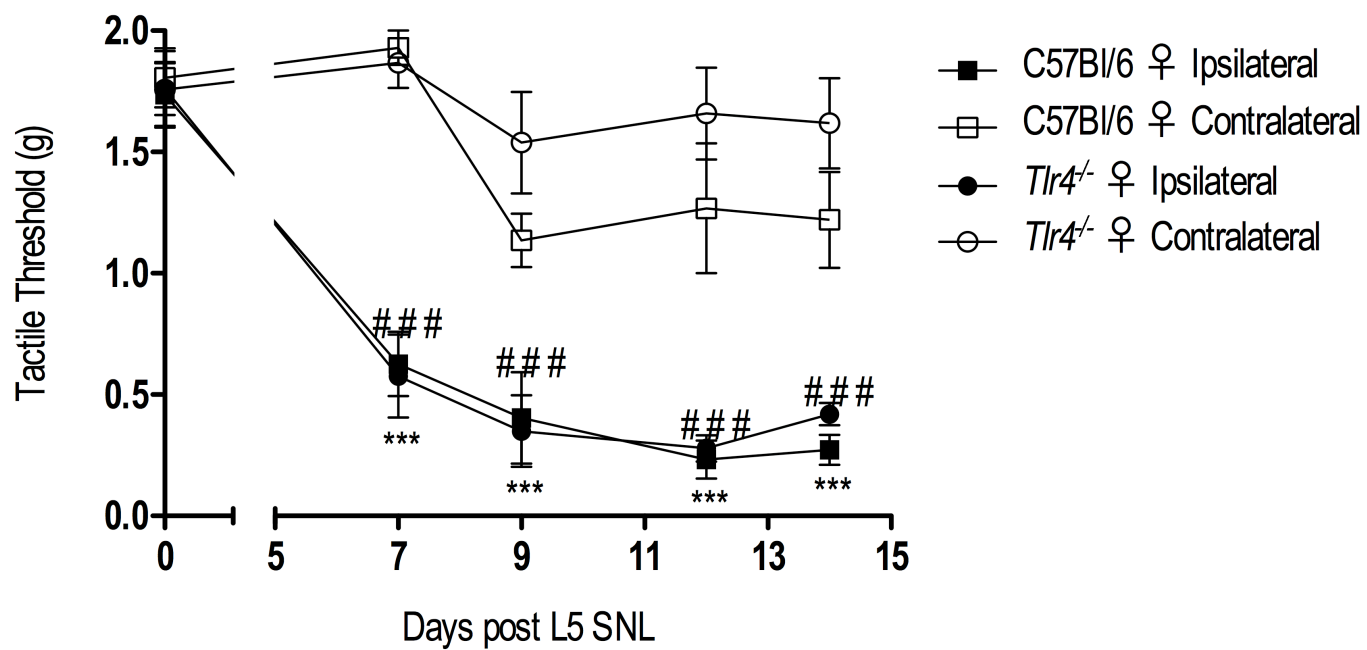

Supplemental Figure 1

Supplement: Additional file 1: Figure S1 — Unilateral TA observed following L5 SNL in C57BL/6 female mice was not reduced in TLR4 signaling-deficient female mice. L5 SNL was performed on female (A) C57BL/6 and (B)Tlr4 -/- mice. Mice were allowed to recover and were tested at days 7, 9, 12, and 14 post-SNL. The solid black line and dashed line represent the C57BL/6 ipsilateral and contralateral thresholds. (A) C57BL/6 mice showed a robust TA in the ipsilateral paw beginning 7 days post-surgery. The (B)Tlr4 -/- mice showed no effect upon the ipsilateral paw tactile threshold following L5 SNL. Data are expressed as mean ± SEM (n = 5 mice/group) and analyzed via 2-way ANOVA, followed by Bonferroni post hoc test to compare each time point to the respective WT C57BL/6 group, ipsilateral or contralateral (###P <0.01 vs. contralateral paw; ***post vs. baseline). [file 1742-2094-10-148-S1.pdf]

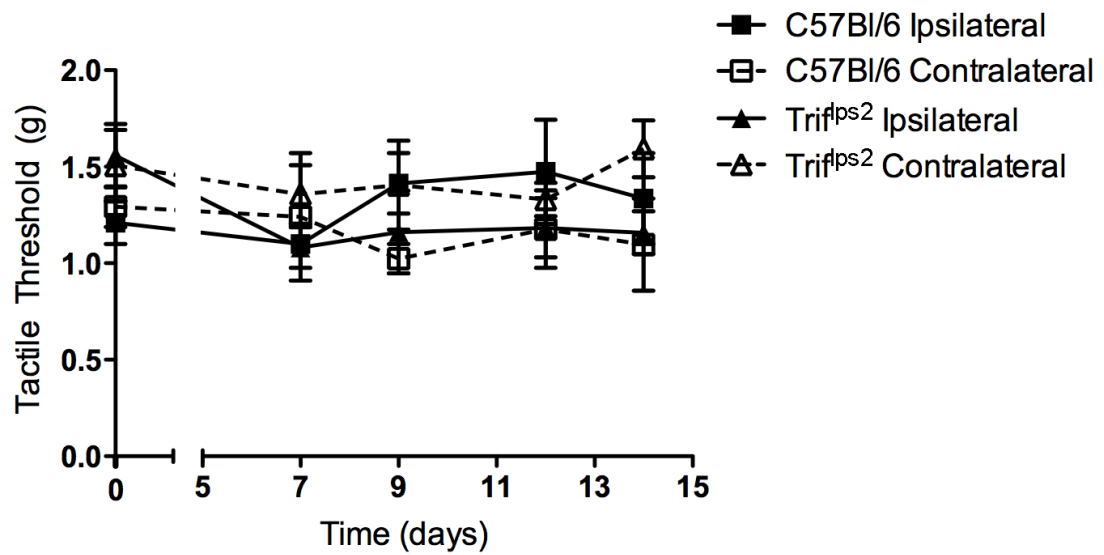

Supplemental Figure 2

Supplement: Additional file 2: Figure S2 — L5 SNL Sham produced no significant effect on tactile thresholds. C57BL/6 and Trif lps2 mice underwent L5 SNL sham surgery and tactile thresholds were measured. There were no significant differences between the tactile thresholds of the four groups as assessed by 1-way ANOVA. [file 1742-2094-10-148-S2.pdf]

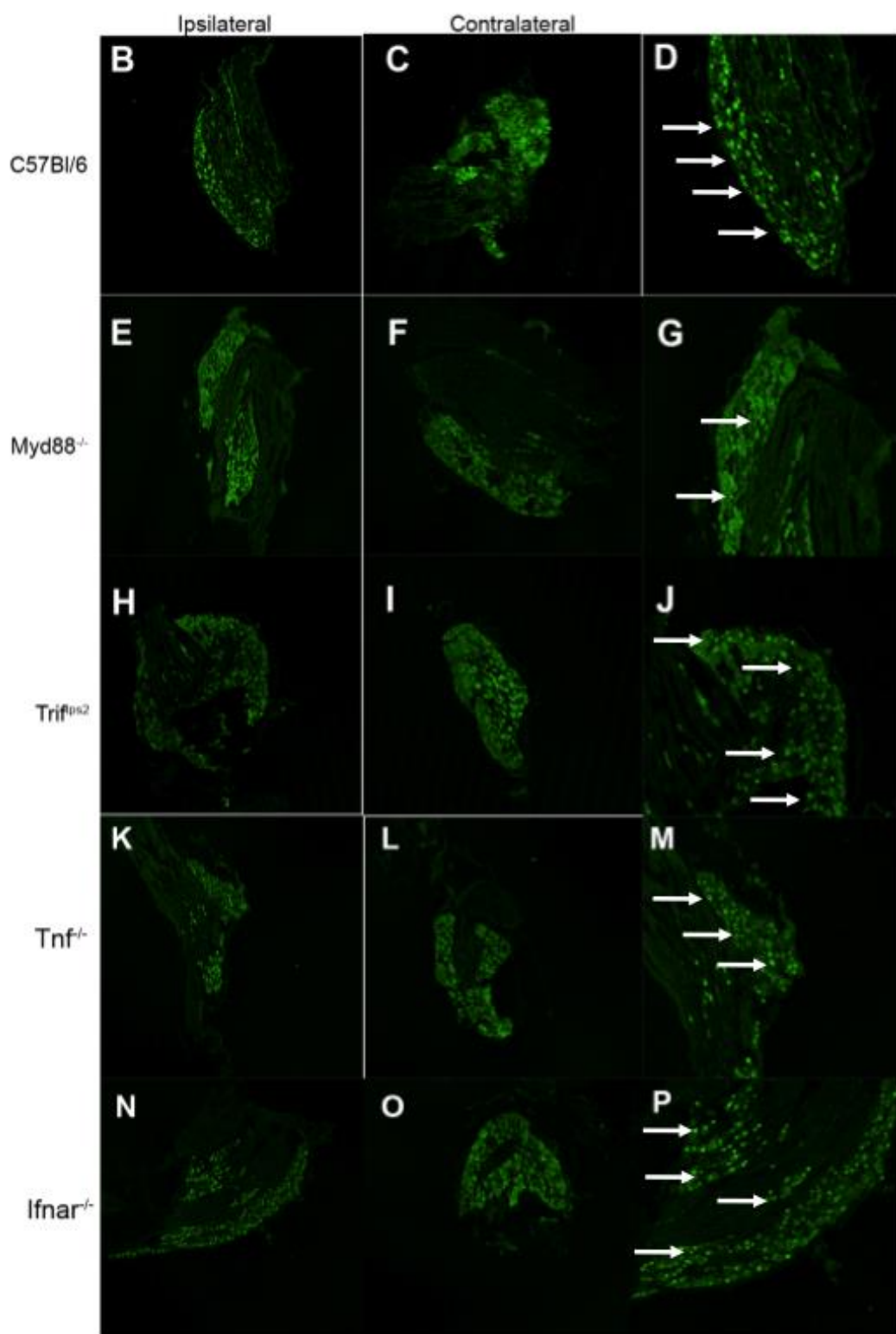

Supplemental Figure 3

Supplement: Additional file 3: Figure S3 — Iba-1 and GFAP immunoreactivity following L5 SNL. At day 14 following L5 SNL, the lumbar region of the spinal cord was harvested and incubated with antibodies against Iba-1 and GFAP. Iba-1 immunoreactivity is visualized with Alexa-488 (green) in the left panel and GFAP with Alexa-594 (red) in the right panel. Quantification for the Iba-1 and GFAP immuno-reactivity is found in Figure 3 and Figure 5. [file 1742-2094-10-148-S3.pdf]

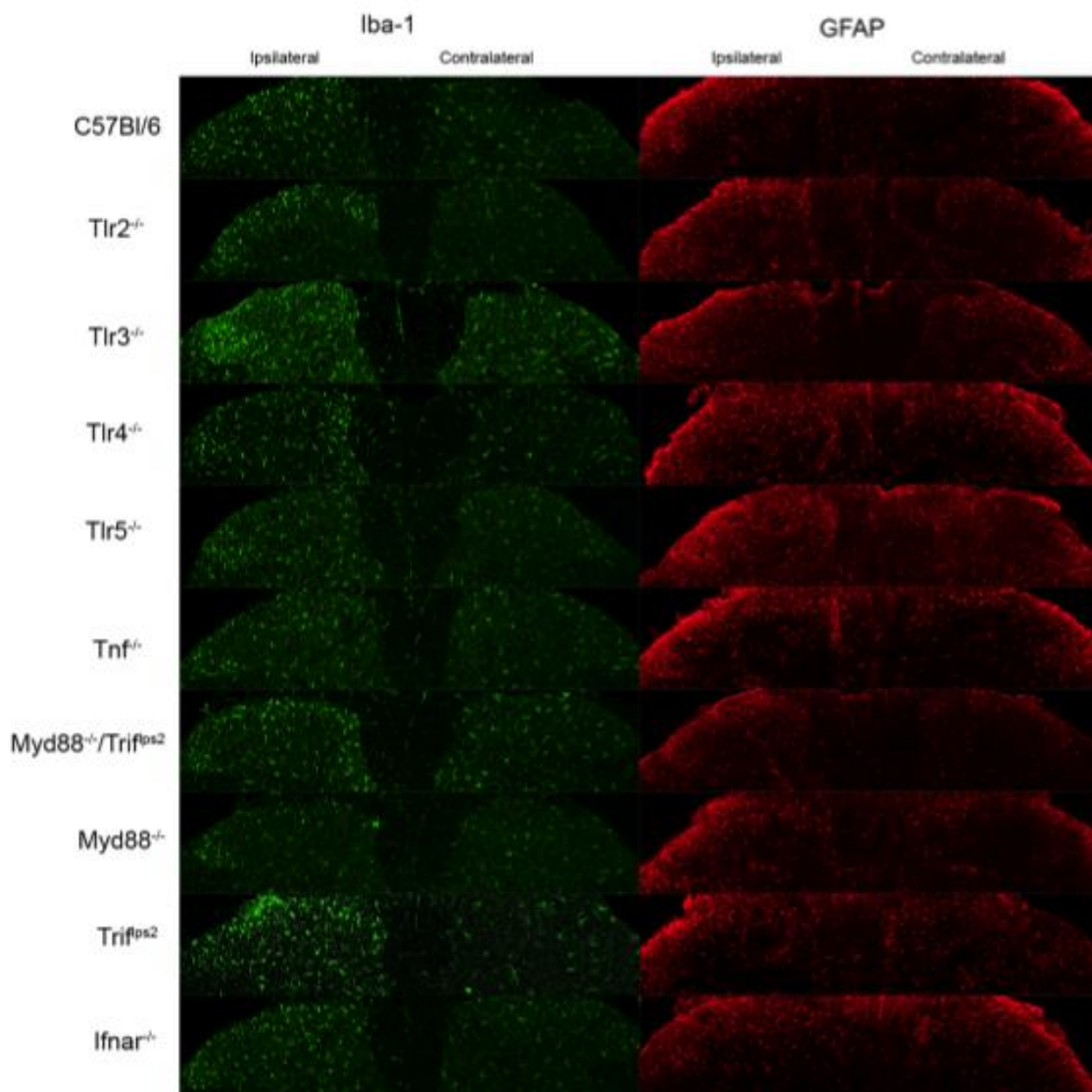

Supplemental Figure 4

Supplement: Additional file 4: Figure S4 — ATF3 immuno-reactivity following L5 SNL. At day 14 following L5 SNL, the L5 right and left DRGs were harvested and incubated with an antibody against ATF3. ATF3 immuno-reactivity is visualized with Alexa-488 (green) and recognized as an intense fluorescent mark in the nuclei region. The white arrows point to examples of ATF3 staining. Quantification for the ATF3 immuno-reactivity is found in Figure 6. [file 1742-2094-10-148-S4.pdf]

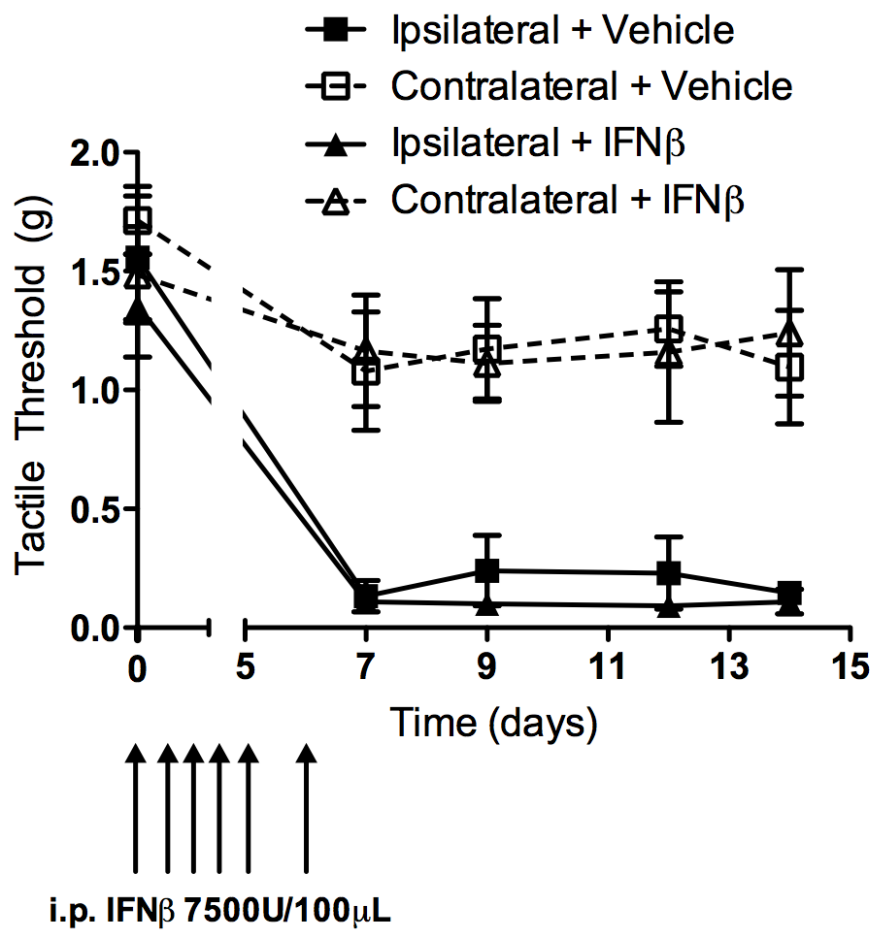

Supplemental Figure 5

Supplement: Additional file 5: Figure S5 — Repetitive treatment with IP IFNβ had no effect on tactile thresholds following L5 SNL. C57BL/6 mice received i.p. IFNβ (7,500 U/100 μL) or vehicle (0.1% BSA) as a pre-treatment on day 0 (before L5 SNL) and days 2, 3, 4, 5, and 6 after L5 SNL. Tactile thresholds were measured on days 7, 9, 12, and 14. No significant differences were found between the i.p. vehicle and i.p. IFNβ groups as assessed by 2-way ANOVA followed by Bonferroni post-hoc test. [file 1742-2094-10-148-S5.pdf]
